# Mechanistic Insights into the Protective Effects of Cryptotanshinone Against CCl_4_-Induced Acute Liver Injury in Mice via Network Pharmacology and Transcriptomics

**DOI:** 10.3390/biom15101449

**Published:** 2025-10-14

**Authors:** Xin Zhang, Qiulin Luo, Yanting Hu, Puyang Gong, Yunsong Zhang, Li Zhang

**Affiliations:** 1 College of Science, Sichuan Agricultural University, Ya’an 625014, China; 2021115002@stu.sicau.edu.cn; 2 College of Pharmacy and Food, Southwest Minzu University, Chengdu 610041, China

**Keywords:** cryptotanshinone, acute liver injury, transcriptomics, network pharmacology

## Abstract

Cryptotanshinone (CPT), the main active compound of *Salvia miltiorrhiza*, is known for its anti-inflammatory, antioxidative, and antifibrotic effects. In this study, the hepatoprotective effect and mechanisms of CPT were explored using transcriptome and network pharmacology. A carbon tetrachloride-induced acute liver injury (ALI) mouse model was established. The anti-ALI effects of different doses of CPT were evaluated by analysis of biochemical indicators, histopathological staining, and immunohistochemical analysis. Combining network pharmacology with transcriptomic analysis revealed therapeutic targets, which were subsequently validated through polymerase chain reaction and Western blotting. CPT (40 mg/kg) treatment significantly reduced the levels of aspartate aminotransferase, alanine aminotransferase, alkaline phosphatase, tumor necrosis factor-α, interleukin-6, and interleukin-1β in model mice and regulated oxidative stress indicators, including malonaldehyde, superoxide dismutase, glutathione, and catalase. MCP-1 protein expression in the liver was inhibited by treatment with CPT. Network pharmacology revealed 72 core targets involved in the treatment of ALI by CPT. By combining transcriptomic data from liver tissue, three key targets—TNF-α, TLR9, and ADORA2B—were identified, along with the TLR, IL-17, and TNF signaling pathways. Furthermore, PCR and Western blot assays revealed that CPT significantly decreased TNF-α, TLR9, and ADORA2B expression levels in the livers of ALI mice. In conclusion, the hepatoprotective effects of CPT may be related to the suppression of TNF-α-, TLR9-, and ADORA2B-mediated inflammation, oxidative stress, and apoptosis. These results provide a foundation for the development of CPT as a potential therapeutic agent for ALI.

## 1. Introduction

Acute liver injury (ALI) is a potential risk factor for multiple serious hepatic diseases that can rapidly evolve into liver failure and even lead to death [1]. ALI can be triggered by factors such as viral hepatitis, alcoholism, toxins, or drugs [2]. ALI, which is potentially fatal, is characterized by widespread hepatocyte necrosis, rapid loss of hepatic function, and high mortality [3]. Once ALI progresses to end-stage liver disease, liver transplantation is regarded as the only effective treatment strategy [4]. Therefore, early prevention and treatment are vital. N-acetylcysteine and glucocorticoids are commonly used drugs for controlling ALI; however, their poor effectiveness and their potential for causing serious adverse reactions have limited their clinical application. Therefore, it is necessary to develop new drugs. In recent years, the discovery of potential anti-ALI drugs from natural compounds of traditional Chinese medicine (TCMs) has attracted widespread attention.

Cryptotanshinone (CPT), a liposoluble diterpenoid derived from *Salvia miltiorrhiza* (SM), has been demonstrated to have various pharmacological activities, including anticancer, anti-inflammatory, antioxidant, antidiabetic, and antiobesity effects [5]. Previous studies have shown that CPT can reduce the activation of caspase-1 activity and the secretion of IL-1β in mouse models of NLRP3 inflammasome-mediated diseases such as endotoxemia syndrome and nonalcoholic steatohepatitis (NASH) [6]. In a tertiary-butylhydroperoxide (tBH)- or d-galactosamine (GalN)-induced primary rat hepatocyte injury model, CPT significantly inhibited lactate dehydrogenase leakage, glutathione (GSH) depletion, lipid peroxidation, and free radical generation [7]. In addition, CPT can promote apoptosis in activated hepatic stellate cells by regulating the endoplasmic reticulum stress pathway in vitro [8]. Although CPT clearly has anti-inflammatory effects and may serve as a promising drug for treating ALI, its underlying mechanism and related targets remain to be elucidated.

Transcriptomics is the comprehensive analysis of the full gene expression profiles of specific tissues or cells under given conditions using high-throughput sequencing technologies (such as the Illumina NovaSeq 6000 platform) [9]. Transcriptomics can be used to identify extensive changes in gene expression levels, which may help to reveal the mechanism of action of specific drugs [10]. Network pharmacology integrates systems biology, bioinformatics, and multidirectional pharmacology to construct a multidimensional interaction network of “drugs–components–targets–diseases”, offering a novel approach to map the interaction networks underlying the effects of TCMs on complex diseases [11,12]. By screening for differentially expressed genes (DEGs) through transcriptomics, conducting interactive verification with the predicted results of network pharmacology is possible. The combination of transcriptomics and network pharmacology has been used to identify the intersection points of DEGs or proteins and has been widely applied in the analysis of the mechanism of action of natural products.

In the present study, a CCl_4_-induced ALI mouse model was established to evaluate the efficacy of CPT through biochemical index analysis, histopathological examination, and immunohistochemical analysis. Network pharmacology, transcriptomics, PCR, and Western blotting analyses were subsequently performed to elucidate the potential mechanisms through which CPT ameliorates ALI, establishing a pharmacological basis for the development of plant-derived therapeutics against ALI.

## 2. Materials and Methods

### 2.1. Materials and Reagents

CPT was purchased from the Nanjing Dige Medical Technology Co., Ltd. in Nanjing, China. (purity ≥ 98%). ALT, AST, and alkaline phosphatase (AKP) kits (Shenzhen Masrui Technology Co., Ltd., Shenzhen, China) and enzyme-linked immunosorbent assay (ELISA) kits for TNF-α, IL-6, and IL-1β (Sichuan Saiinst Biotechnology Co., Ltd., Leshan, China) were used. Malonaldehyde (MDA), SOD, and GSH levels were assessed using assay kits (Nanjing Jiancheng Institute of Bioengineering Co., Ltd., Nanjing, China). A CAT kit (Elabscience Biotechnology Co., Ltd., Wuhan, China), dinitrobenzoic acid (DNB) (Lanjieke Technology Co., Ltd., Beijing, China), xylene, hydrochloric acid, anhydrous ethanol, and ammonia water (ChengDu Chron Chemicals Co., Ltd., Chengdu, China) were also used.

### 2.2. Animals and Experimental Design

Sixty SPF-grade male C57BL/6 mice (weighing 18–22 g) were purchased from Chengdu Dashuo Experimental Animal Co., Ltd. in Chengdu, China. (Production License No.: SYXK (Chuan) 2020-0030). All experimental procedures were approved by the Animal Ethics Committee of Sichuan Agricultural University (No. 20240089). The mice were placed in an environment of approximately 22 ± 2 °C, with a 12 h light/dark cycle, and were fed standard food and tap water at will. The mice were randomly divided into six groups (10 mice per group): the control group, model group, low-dose CPT group (CPT-L, 10 mg/kg), medium-dose CPT group (CPT-M, 20 mg/kg), high-dose CPT group (CPT-H, 40 mg/kg), and silymarin (SIL) group (84 mg/kg). The establishment method of the ALI model follows the approach outlined in our laboratory’s prior research [13]. Briefly, with the exception of those in the control group that were injected with an olive oil solvent without CCl_4_, all the other mice were intraperitoneally injected with 10% CCl_4_ (diluted in olive oil and filtered through a 0.22 μm membrane prior to administration) at a dose of 2.5 mL/kg [14,15]. At 6 h of postmodeling, the treatment groups received their first intravenous injection of CPT at the corresponding doses, while the control and model groups were administered an equal volume of PBS. The same treatment regimen was repeated on day 3. The animals were sacrificed on day 4, and blood and tissue samples were collected. A subset of tissue samples was cryopreserved at −80 °C for biochemical assays, while the remaining samples were stored in 4% formalin buffer for histological examination.

### 2.3. Determination of Biochemical Indicators

Serum and liver tissue samples from six mice were randomly selected from each group. Detection kits were used, and the levels of ALT, AST, AKP, TNF-α, IL-6, IL-1β, MDA, SOD, GSH, and CAT were measured in strict accordance with the manufacturer’s instructions.

### 2.4. Liver Histopathological Examination

Fresh liver tissues were removed immediately, fixed in 4% formalin, embedded in paraffin, cut into slices, dewaxed, hydrated, and finally stained with hematoxylin and eosin (HE). For histopathological evaluation, the tissues were sliced into conventional rotary paraffin wax with a thickness of 4 μm, subjected to HE staining, and photographed under a microscope to observe histopathological changes.

### 2.5. Immunohistochemical Staining

Liver tissue samples (n = 3 per group) were processed into paraffin-embedded sections. The sections were dewaxed in xylene (10 min), subjected to antigen retrieval by heating in citrate buffer, and then washed with PBS. Endogenous peroxidase activity was blocked with 10% hydrogen peroxide (10 min, room temperature), followed by treatment with goat serum (10–15 min). The sections were incubated with primary antibodies overnight at 4 °C, followed by incubation with secondary antibodies (37 °C, 20 min). DAB substrate was used for chromogenic detection. The sections were counterstained with hematoxylin, differentiated in hydrochloric acid-alcohol, dehydrated, cleared in xylene, and mounted with neutral resin. MCP-1 protein expression was observed under a microscope.

### 2.6. Network Pharmacology Research

#### 2.6.1. Screening of Constituents and Disease-Related Targets

On the basis of the chemical entity retrieval module of the TCMs Systems Pharmacology (TCMSP) database (https://www.91tcmsp.com/#/database (accessed on 18 March 2024)), an exact search was performed using “Chemical name” as the query field and “Cryptotanshinone” as the keyword. The system screened and generated the original target dataset for CPT. PubChem (https://pubchem.ncbi.nlm.nih.gov/ (accessed on 18 March 2024)) was used to construct the canonical Simplified Molecular Input Line Entry System (SMILES) of CPT. The Swiss Target Prediction database (http://www.swisstargetprediction.ch/ (accessed on 18 March 2024)) and the TCMSP database were used to predict compound-related targets. The keyword “acute liver injury” was input into the Human Gene (GeneCards) Database (https://www.genecards.org/ (accessed on 18 March 2024)) and Online Mendelian Inheritance in Man (OMIM) database (https://omim.org/ (accessed on 18 March 2024)) to collect disease targets.

#### 2.6.2. Construction of the Protein-Protein Interaction (PPI) Network

The Venny 2.1 platform (https://bioinfogp.cnb.csic.es/tools/venny/index.html (accessed on 20 March 2024)) was used to generate the intersection targets of CPT-ALI, which represented the potential target set of CPT in the treatment of ALI. The selected intersection targets were imported into the STRING database (https://cn.string-db.org/ (accessed on 20 March 2024)), and the species “*Homo sapiens*” was selected to export the tsv file of the intersection genes in the PPI network. The obtained data were subsequently input into Cytoscape software, version 3.7.2, to construct a PPI map.

#### 2.6.3. Gene Ontology (GO) and Kyoto Encyclopedia of Genes and Genomes (KEGG) Enrichment Analyses

GO and KEGG pathway enrichment analyses of the core targets were performed using the Database for Annotation, Visualization, and Integrated Discovery (DAVID 6.8). At the level of GO annotation, enrichment analysis was performed for three ontological dimensions (False Discovery Rate (FDR) < 0.05): molecular function (MF), biological process (BP), and cellular component (CC). A *p* < 0.05 was considered to indicate statistical significance, and the top 20 items significantly enriched according to the Wei Sheng Xin platform (https://www.bioinformatics.com.cn/ (accessed on 21 March 2024)) are displayed.

### 2.7. Transcriptomic Sequencing and Data Analysis

Total RNA was extracted from random liver tissue samples (n = 6) from the control, model, and CPT-H groups, and its purity was verified. cDNA libraries were constructed, and RNA quality was assessed according to standardized sequencing protocols on the Illumina NovaSeq 6000 platform [16]. Differentially expressed genes (DEGs) were screened using the criteria of |log_2_FC| > 1.5, *p* < 0.05, and FDR < 0.05. These DEGs were visualized through volcano plots, Venn diagrams, and heatmaps to illustrate expression patterns and overlaps across groups. GO and KEGG pathway enrichment analyses were performed using the DAVID database, followed by visualization of the significantly enriched terms and pathways.

### 2.8. Quantitative Real-Time PCR

Total RNA was extracted from random liver tissue samples using the TRIzol method according to the manufacturer’s instructions and subsequently reverse transcribed into cDNA using a reverse transcription kit. The mRNA expression levels of *TNF*, *TLR9*, and *ADORA2B* were quantified via qRT-PCR. β-Actin was used as the internal reference gene, and relative gene expression was calculated using the 2^−ΔΔCT^ method. The primer sequences for the analyzed target genes are listed in Table 1.

### 2.9. Western Blotting

First, random liver tissue samples from the control, model, and CPT-H groups were rinsed three times with PBS and then minced and homogenized in a 10× volume of RIPA lysis buffer. After centrifugation (12,000 rpm, 10 min, 4 °C), protein concentrations were quantified using a BCA assay kit. Next, the proteins were separated by SDS-PAGE and electrophoresis onto PVDF membranes. Third, the membranes were blocked with 5% skim milk in TBST (1 h, RT) and then incubated overnight with primary antibodies (1:1000 dilution, 4 °C). Following three PBST washes (5 min each), the membranes were incubated with HRP-conjugated secondary antibodies (1:10,000 dilution, 1 h). Finally, signal detection was performed using an advanced ECL kit, and chemiluminescent images were captured using a digital imaging system.

### 2.10. Statistical Analysis

Data analysis and graph generation were performed using GraphPad Prism 8.0.2 software. The Shapiro–Wilk test was used to verify normality, and the Brown–Forsythe test was used to verify homogeneity of variance. The data that met the above criteria were analyzed using *t*-tests and one-way analysis of variance (ANOVA) and are all presented in the form of mean ± standard deviation (SD). If the data did not meet the criteria, non-parametric tests were used, and the results are presented in the form of median and interquartile ranges. *p* < 0.05 was considered statistically significant.

## 3. Results

### 3.1. Effects of CPT on Mice with ALI Induced by CCl_4_

**Figure 1:**
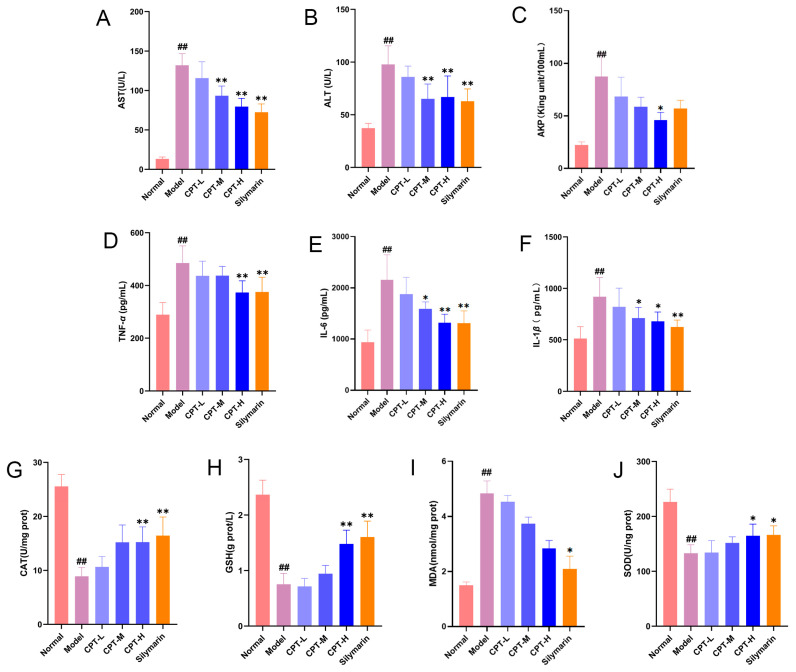
CPT alleviates hepatic injury, inflammation, and oxidative stress in CCl_4_-induced ALI mice. (**A**) AST, (**B**) ALT, (**C**) AKP, (**D**) TNF-α, (**E**) IL-6, (**F**) IL-1β, (**G**) CAT, (**H**) GSH, (**I**) MDA, and (**J**) SOD levels in the experimental groups (n = 6). Except for the MDA data, which are expressed as the median and interquartile ranges, all other data are expressed as the mean ± standard deviation (SD). Significant differences are indicated as follows: ^##^ *p* < 0.01 vs. the normal group; * *p* < 0.05, ** *p* < 0.01 vs. the model group.

Compared with those in the control group, serum levels of AST, ALT, and AKP were significantly elevated in the model group, indicating that the intraperitoneal injection of CCl_4_ successfully induced ALI in mice (*p* < 0.01). In contrast to the model group, the CPT-H and the positive control drug SIL groups markedly reduced serum AST, ALT, and AKP levels in the model group (*p* < 0.05). However, no significant improvement was observed in the CPT-L group (Figure 1A–C). These results suggest that CPT exerts a significant protective effect against CCl_4_-induced liver injury.

In addition, in the CCl_4_-induced mouse ALI model, a pronounced inflammatory response was observed. Serum levels of TNF-α, IL-1β, and IL-6 were significantly elevated in the model group compared with those in the control group (*p* < 0.01). CPT-H and SIL markedly reduced the upregulation of these proinflammatory cytokines (*p* < 0.05, *p* < 0.01). CPT m attenuated IL-1β and IL-6 levels (*p* < 0.05), whereas CPT-L did not significantly ameliorate these effects (Figure 1D–F). These findings suggest that the anti-inflammatory effect is one of the key mechanisms underlying the hepatoprotective activity of CPT. Compared with those in the control group, the levels of CAT, GSH, and SOD in the model group significantly decreased, indicating that CCl_4_ induced liver oxidative stress (*p* < 0.01). Following treatment with CPT-H and SIL, the CAT, GSH, and SOD levels significantly increased (*p* < 0.05), demonstrating that CPT alleviated oxidative stress in the liver tissues of mice with ALI (Figure 1G–J).

**Figure 2:**
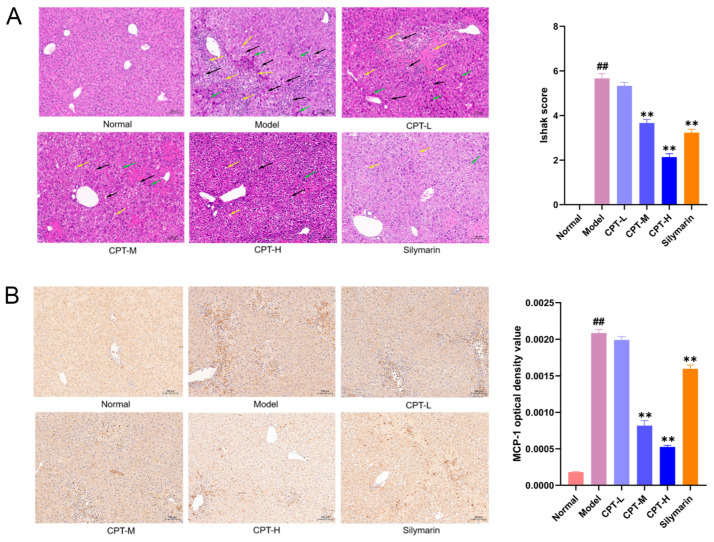
Pathology evaluation of the ability of CPT to ameliorate CCl_4_-induced ALI in mice: (**A**) Representative image of H&E staining (200×). Quantitative analysis of liver injury was performed using the Ishak scoring system. (**B**) Regulatory effects of CPT on MCP-1 expression in the liver tissues of ALI mice (200×). Data are expressed as the mean ± standard deviation (SD). (n = 3). Significant differences are indicated as follows: ^##^ *p* < 0.01 vs. the normal group; ** *p* < 0.01 vs. the model group (black arrowheads: inflammatory cell infiltration; green arrowheads: hepatocyte necrosis; yellow arrowheads: fat vacuoles).

Histopathological evaluation of liver tissues revealed distinct differences among the groups (Figure 2A). In the control group, the hepatic lobular architecture remained intact, with hepatocytes arranged in orderly radial cords. No significant necrosis, inflammatory infiltration, or lipid vacuolization was observed. In contrast, the model group exhibited extensive hepatocyte necrosis, and slight bleeding was observed in the necrotic area of the cells, accompanied by massive inflammatory cell infiltration (shown as black arrowheads). Notably, treatment with CPT-M and CPT-H, as well as SIL, significantly reduced hepatocyte necrosis, preserved lobular architecture, reduced inflammatory cell infiltration, attenuated lipid vacuolization, and rarely resulted in hemorrhage.

Monocyte chemoattractant protein-1 (MCP-1), a key chemokine involved in the induction and chemotactic recruitment of inflammatory cells, is overexpressed during immune dysregulation and inflammatory responses [17]. As demonstrated in Figure 2, intraperitoneal administration of CCl_4_ significantly upregulated liver MCP-1 expression in mice. Notably, treatment with CPT-M and CPT-H and the positive control drug markedly suppressed this CCl_4_-induced MCP-1 overexpression. These findings suggest that CPT ameliorates hepatic injury by attenuating MCP-1-mediated inflammatory cascades within the liver microenvironment (Figure 2B).

### 3.2. Network Pharmacology Analysis

#### 3.2.1. Prediction of Candidate Targets Related to CPT and ALI

Through a search of the TCMSP database, 30 component targets of CPT were initially identified. Following gene name standardization, one target lacking a standardized gene name and nine targets not identified in human genes were excluded, resulting in 20 valid targets. From the SwissTargetPrediction database, 100 CPT-related targets were identified. After filtering with the criterion of “probability > 0” and removing duplicates, 67 targets remained. Merging the potential targets from both databases and eliminating redundancies yielded a total of 82 targets.

A total of 8736 disease targets associated with ALI were subsequently identified through the GeneCards and OMIM databases. Using Venny 2.1, intersection analysis between CPT component targets and ALI disease targets revealed 73 overlapping targets, which were predicted as potential therapeutic targets for CPT against ALI.

#### 3.2.2. PPI Network Analysis

**Figure 3:**
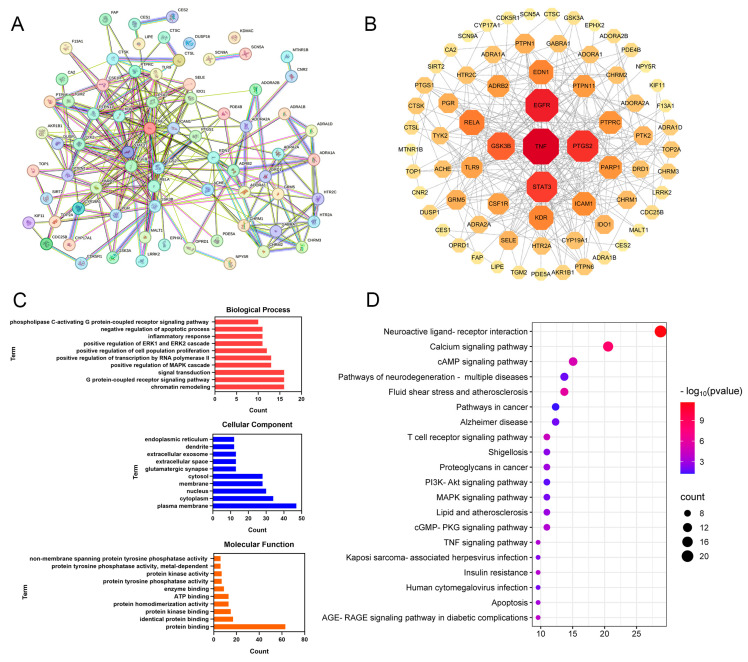
Network pharmacology analysis of CPT targets against ALI: (**A**,**B**) PPI network analysis. (**C**) GO functional enrichment analysis. (**D**) KEGG pathway enrichment analysis.

The PPI network comprised 73 nodes and 286 edges, with an average node degree of 7.84 (Figure 3A,B). The targets were ranked by their degree values, as illustrated in Figure 3B. In the PPI network, the larger the dimensions and the deeper the hues are, the more important the proteins are. Ultimately, CPT may exert its anti-ALI effects through key proteins such as TNF, EGFR, PTGS2, STAT3, and GSK3B.

#### 3.2.3. GO and KEGG Enrichment Analyses

GO and KEGG enrichment analyses were conducted on 73 core targets using the DAVID database (*p* < 0.05). As shown in Figure 3C, the 10 terms with the greatest degree of enrichment in BP, CC, and MF were screened out. The key enriched terms included chromatin remodeling, G protein-coupled receptor signaling pathway, cytoplasmic membrane, cytoplasm, nucleus, protein binding, and protein kinase binding.

KEGG pathway enrichment analysis revealed 59 significantly enriched pathways (*p* < 0.05). As shown in Figure 3D, the top 20 pathways were annotated and primarily involved the neuroactive ligand-receptor interaction, calcium signaling pathway, cAMP signaling pathway, T-cell receptor signaling pathway, and PI3K-Akt signaling pathway. These results suggest that CPT may ameliorate ALI through multitarget regulation across multiple pathways.

### 3.3. Transcriptome Analysis to Assess the Effects of CPT in Mice with ALI

**Figure 4:**
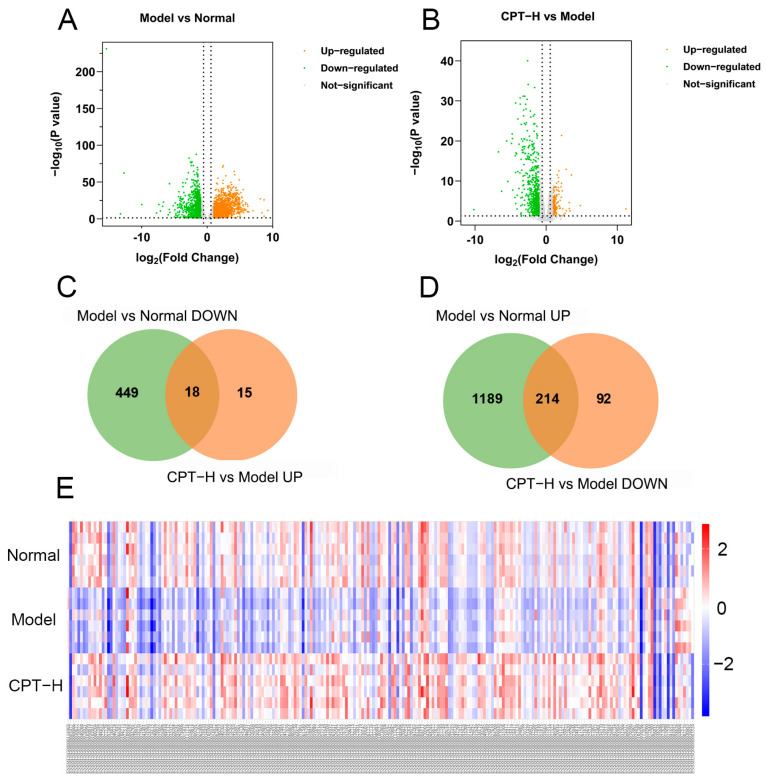
Regulation analysis of DEGs in ALI mouse liver under CPT intervention: (**A**) volcano plot of DEGs between the model and normal groups; (**B**) volcano plot of DEGs between the CPT-H and model groups; (**C**) intersection of downregulated genes in the model group and upregulated genes in the CPT-H group; (**D**) intersection of upregulated genes in the model group and downregulated genes in the CPT-H group; (**E**) hierarchical clustering heatmap of 232 DEGs.

On the basis of the sequencing results of gene expression levels in each group, the screening criteria for DEGs were | log2 (fold change) | >1.5, *p* < 0.05, and FDR < 0.05. A total of 1870 DEGs were identified in the model group compared with the normal group, of which 1403 DEGs were upregulated and 467 DEGs were downregulated. After treatment with CPT-H, 339 DEGs were identified, of which 33 DEGs were upregulated and 306 DEGs were downregulated (Figure 4). The green and orange dots in the volcano map represent the genes whose expression levels were downregulated and upregulated, respectively. The more the data points deviate from the midline, the more significant the difference in expression levels (Figure 4A,B). The results of heatmap clustering also revealed significant differences in gene expression between the model group and the normal group or between the CPT-H group and the model group (Figure 4E).

Furthermore, as shown in Figure 4C,D, further analysis revealed that 232 DEGs were significantly reversed after CPT treatment, suggesting that these DEGs were not only involved in the liver injury process caused by CCl4 but may also mediate the anti-ALI efficacy of CPT.

### 3.4. GO and KEGG Pathway Analyses of CPT-Regulated DEGs in ALI

**Figure 5:**
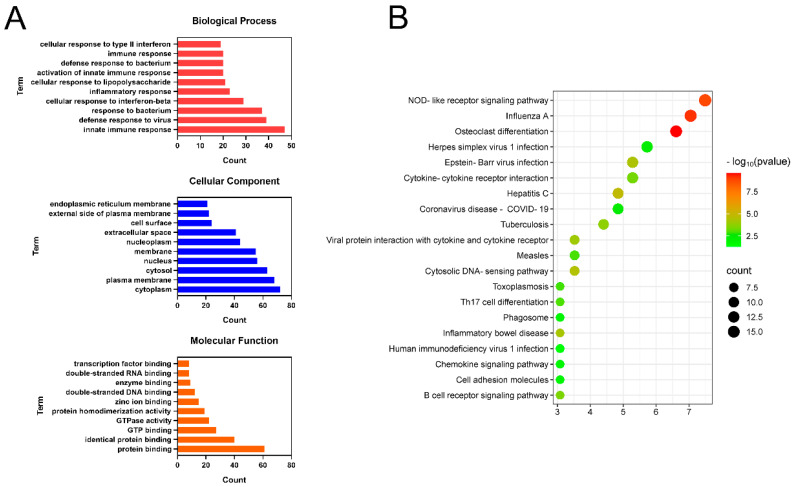
Functional enrichment analysis of DEGs: (**A**) GO enrichment analysis of DEGs; (**B**) KEGG pathway enrichment analysis of DEGs.

To further clarify the biological functions involved in the anti-liver injury effects of CPT, GO function and KEGG pathway enrichment analyses were conducted on the 232 DEGs screened out. GO terms were classified into three categories (Figure 5A). The main BPs enriched by DEGs included innate immune responses, inflammatory responses, and cellular responses to interferon-beta. CCs included mainly the cytoplasm, plasma membrane, and cytosol. MFs included mainly protein binding, GTO binding, and enzyme binding. As shown in Figure 5B, the results of the KEGG enrichment analysis indicated that the DEGs were enriched mainly in NOD-like receptor signaling pathways, osteoclast differentiation, and cytokine–cytokine receptor interactions.

### 3.5. Integrated Network Pharmacology and Transcriptomics Analysis

An integrative network pharmacology and transcriptomics analysis was conducted to elucidate the molecular mechanisms underlying the hepatoprotective effects of CPT. Network pharmacological analysis revealed 73 potential therapeutic targets of CPT against ALI, while transcriptomic screening revealed 232 DEGs. Intersection analysis revealed three overlapping genes, namely, tumor necrosis factor (*TNF*), Toll-like receptor 9 (*TLR9*), and adenosine A2B receptor (*ADORA2B*). Furthermore, a total of 14 common signaling pathways, predominantly the TLR signaling pathway, IL-17 signaling pathway, and TNF signaling pathway, were identified through network pharmacology and transcriptomics studies. Notably, *TNF*-α was significantly enriched across multiple inflammation-associated pathways (TLR, IL-17, and TNF pathways), whereas *TLR9* was specifically enriched in the TLR pathway. These results suggest that CPT may improve ALI through regulation of the TLR, IL-17, and TNF signaling pathways mediated by key genes such as *TNF*, *TLR9*, and *ADORA2B*.

### 3.6. Verification of the mRNA and Protein Expression Levels of TNF, TLR9, and ADORA2B

**Figure 6:**
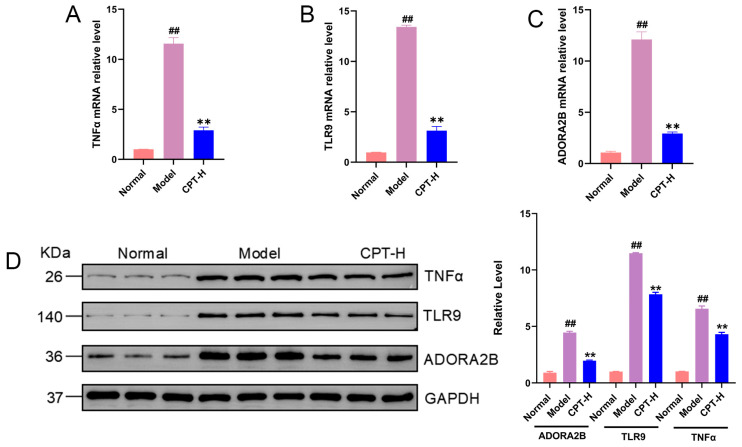
Effects of CPT on TNF, TLR9, and ADORA2B expression in the livers of ALI mice. (A) TNFα, (B) TLR9 and (C) ADORA2B mRNA relative levels in the experimental groups. (D) Protein expression levels of TNF, TLR9, and ADORA2B. Data are expressed as the mean ± standard deviation (SD) (n = 3). Significant differences are indicated as follows: ^##^ *p* < 0.01 vs. the normal group; ** *p* < 0.01 vs. the model group. Western blot original images can be found in Figure S1.

To further validate the gene regulatory effects of CPT on liver tissues in ALI mice, a comparative analysis of the mRNA and protein expression levels of the key genes TNF, TLR9, and ADORA2B was conducted across the treatment groups, control group, and model group. As shown in Figure 6, compared with those from the control group, liver tissues from the model group exhibited significantly upregulated mRNA and protein expression levels of TNF, TLR9, and ADORA2B (*p* < 0.01). Strikingly, CPT-H treatment markedly suppressed the CCl_4_-induced overexpression of these genes, with reductions observed in mRNA abundance and protein synthesis. These experimental findings align with prior transcriptomic profiling data.

## 4. Discussion

Owing to the similarity between ALI induced by CCl_4_ in mice and acute chemical liver injury in humans, the ALI mouse model is widely used to investigate potential therapeutic strategies [18]. CCl_4_ is metabolized by various cytochromes (such as CYP2E1) to generate trichloromethyl radicals (CCl_3_), which produce peroxyl radicals (CCl_3_OO). These radicals are highly reactive and can attack lipids, proteins, and nucleic acids, causing lipid peroxidation, protein damage, and DNA damage [19]. Eventually, this leads to the disruption of membrane structure and functional integrity, an increase in the cytoplasmic Ca^2+^ concentration, and, consequently, liver cell death [20]. Moreover, CCl_4_ induces the liver to release various inflammatory factors, which regulate cell survival, apoptosis, and fibrosis through complex signaling pathways, thereby exacerbating liver injury [21]. The mechanism of liver injury caused by CCl_4_ in animal models is relatively clear, and the preparation is simple; therefore, this model was adopted in this study to explore the anti-ALI effect and mechanism of CPT.

ALT, AST, and AKP, which are essential liver enzymes and important liver function markers, exhibit dynamic changes in serum levels with distinct pathological implications [22]. ALT and AST are located primarily in the cytoplasm and mitochondria of hepatocytes. When the integrity of the hepatocyte membrane is compromised, these enzymes are released from cells and can convert alanine and aspartic acid into pyruvate or oxaloacetic acid. Their concentration elevation positively correlates with the extent of hepatocellular necrosis [23]. AKP, an important immune factor, plays a crucial role in the dephosphorylation of proteases and typically has abnormally increased activity in metabolic and immune disorders [24]. In this study, the CCl_4_-induced model group had significantly increased serum ALT, AST, and AKP levels and damaged hepatocytes, whereas the CPT treatment group had significantly decreased AST, ALT, and AKP levels, indicating that CPT can protect the liver and maintain stable liver function.

Oxidative stress refers to increased intracellular levels of reactive oxygen species (ROS), which can lead to damage to lipids, proteins, and DNA [25]. Excessive radicals accelerate the degradation of cell membranes, disrupt the balance between oxidation and antioxidation, and damage cellular integrity, ultimately causing damage to the organism [26]. CCl_4_ induces severe hepatocyte necrosis by generating reactive oxygen species (ROS), leading to a decrease in SOD levels because of excessive consumption [27]. MDA, the terminal product of ROS-mediated lipid peroxidation, reflects the extent of cellular oxidative damage. The oxidative stress defense system (which comprises SOD, GSH, and CAT) plays a critical role in counteracting elevated lipid peroxides during liver injury [28]. SOD and GSH typically synergize with CAT in the cytoplasm to remove excess ROS, thereby protecting the liver from excessive oxidative stress [29]. Following CCl_4_ exposure, inflammatory pathways are activated, representing a critical pathological mechanism in liver injury. Excessive production of key proinflammatory cytokines (such as TNF-α, IL-6, and IL-1β) exacerbates liver damage [30]. TNF-α promotes inflammatory responses by stimulating hepatic production of acute phase proteins, suppressing the synthesis of albumin, and synergizing with IL-6 [31,32]. Concurrently, IL-1β induces acute-phase reactant synthesis in hepatocytes, promotes neutrophil infiltration and activation, and amplifies inflammatory liver injury [33]. Consequently, therapeutic strategies targeting oxidative stress and inflammation are considered effective approaches for treating CCl_4_-induced liver injury. The results demonstrated that CPT treatment increased the activities of SOD, GSH, and CAT to varying degrees, and alleviated liver inflammation by reducing the levels of the inflammatory factors TNF-α, IL-6, and IL-1β, indicating that the hepatoprotective effects were mediated through the inhibition of oxidative stress and suppression of inflammation mechanisms.

Kupffer cells (KCs) and monocyte-derived macrophages (MoMFs) located in the hepatic macrophage compartment play pivotal roles in modulating inflammation and tissue repair in mice. During the early phase of ALI, the number of KCs is reduced while MoMFs extensively infiltrate the liver, and the opposite trend is observed during the repair process. Activated hepatic stellate cells (HSCs) interact with MoMFs in the necroinflammatory and early repair phases, promoting hepatic regeneration [34]. MCP-1, a key CC chemokine critical for monocyte/macrophage recruitment to injury sites, is significantly upregulated during liver damage pathology [35,36]. In this study, HE staining revealed preserved lobular architecture, normalized nuclear-cytoplasmic ratios, and reduced inflammatory infiltration in the CPT groups. Furthermore, IHC confirmed that CPT might restore liver structure by enhancing KC/MoMF-mediated repair, promoting hepatocyte recovery, and suppressing MCP-1 expression.

At the transcriptional level, the gene expression profiles of humans and mice are generally conserved, although the degree of conservation varies across tissues and compared genes. Due to the close evolutionary relationship between mice and humans, and owing to their numerous characteristics that facilitate manipulation, mice have been used as animal models in biomedical research for over 50 years to study mammalian development, disease, and drug testing [37]. Network pharmacology and transcriptomics demonstrated that CPT may regulate the TNF-α, TLR9, and ADORA2B proteins, which in turn affect the TLR, IL-17, and TNF pathways. The TLR signaling pathway is a critical mediator of innate immunity. Key receptors in this pathway (including TLR3, TLR4, and TLR9) recognize pathogen-associated molecular patterns (PAMPs) derived from microbes and damage-associated molecular patterns (DAMPs), triggering immune cell activation and initiating innate immune responses [38]. Research by Xin-Xing Li et al. on the anti-inflammatory mechanisms of CPT in mouse macrophages revealed that CPT suppresses lipopolysaccharide (LPS)-induced TLR4 and MyD88 expression in RAW264.7 macrophages by modulating the TLR4/MyD88 signaling pathway, thereby mediating its anti-inflammatory effects [39]. TLR9, which is localized in endosomes, recognizes DAMPs such as mitochondrial DNA (mtDNA), transcription factor A mitochondrial (TFAM), and ATP, which critically regulate inflammatory environments in human diseases. In hepatocellular carcinoma (HCC), mtDNA released from the extracellular matrix activates TLR9 signaling, triggering inflammatory responses [40]. In lung cancer, miR-574-5p overexpression is correlated with tumor invasion and metastasis. CPT modulates miR-574-5p expression via promoter methylation and targets protein tyrosine phosphatase receptor type U (PTPRU) to influence TLR9 signaling, thereby affecting apoptosis and improving lung cancer [41,42]. However, the role of TLR9 in liver pathologies remains poorly understood, and the mechanism through which CPT regulates TLR9-mediated inflammation to ameliorate liver injury requires further exploration.

The TNF signaling pathway, which is mediated primarily by TNF-α and its receptors TNFR1/TNFR2, is a critical intracellular cascade involved in inflammatory regulation. In ALI, TNF-α overexpression activates this pathway, driving NF-κB activation and the subsequent release of proinflammatory cytokines, which exacerbate hepatocyte apoptosis, necrosis, and liver damage [43]. TNF-α, a pivotal cytokine in hepatic inflammation and injury, drives inflammatory responses, apoptosis, and oxidative stress [44]. TNF-α plays a pivotal and multifaceted role in NASH pathogenesis. By acting as a key proinflammatory cytokine, TNF-α drives critical disease processes, including liver inflammation, cell death, and fibrosis, through its receptor-mediated downstream signaling pathways. Its expression is regulated by upstream factors such as NF-κB during NASH progression. Because TNF-α is a causal factor that significantly contributes to disease progression, it represents a promising therapeutic target [45]. Furthermore, Zhining Gao et al. reported that TNF-α initiates the apoptosis of hepatocytes through TNFR1-dependent activation of the FADD/caspase-8 cascade and mitochondrial cytochrome c release, leading to caspase-9/caspase-3 activation [44]. In a chronic obstructive pulmonary disease (COPD) mouse model, CPT alleviated lung injury by activating the Keap1/Nrf2 pathway to reduce ROS levels, suppress NF-κB signaling, and lower TNF-α concentrations in pulmonary tissue serum, thereby mitigating inflammation [46]. The results of this study indicate that CPT may protect against ALI by targeting TNF-α in TNF signaling pathways, suppressing inflammation, attenuating apoptosis, and modulating oxidative stress.

ADORA2B, a member of the adenosine receptor family and a G protein-coupled receptor (GPCR), is activated by extracellular adenosine to regulate inflammation, hypoxia adaptation, and immune responses [47]. Under ischemic or hypoxic conditions, ADORA2B promotes erythropoiesis, vascular remodeling, and tissue protection, whereas in chronic inflammatory diseases, it modulates cytokine release and immune cell activity [48]. ADORA2B signaling is essential for mediating the anti-inflammatory effects of CXCR4/CXCR7 inhibition in peritonitis-associated sepsis. This mechanism, dependent on ADORA2B, has been confirmed both in vivo and in vitro [49]. Tiago F Granja et al. reported that during liver ischemia/reperfusion (IR) injury, the volatile anesthetic sevoflurane exhibits anti-inflammatory and tissue-protective effects by increasing the transcription and expression of ADORA2B in wild-type liver tissues [50]. The above studies suggest that ADORA2B may play a role in controlling the inflammatory response during the amelioration of ALI by CPT.

In addition, the results of transcriptomics and network pharmacology collectively revealed that the IL-17 signaling pathway may mediate the anti-ALI efficacy of CPT. Interleukin-17A (IL-17A) is an isoform of the IL-17 family of cytokines. The IL-17 signaling pathway, mediated by the cytokine IL-17A and its receptor IL-17 receptor A, promotes inflammation, fibrosis, and HCC progression in alcohol-related liver disease (ALD) and NASH [51]. In an ulcerative colitis model, CPT reduced the proportion of CD4IL-17ATh17 cells in the spleen and mesenteric lymph nodes, suppressed IL-17A levels in colonic tissues, and inhibited TGF-β/IL-6-driven Th17 cell differentiation, thereby decreasing proinflammatory cytokine secretion (IL-17A and TNF-α) [52]. This evidence provides guidance for in-depth research on the precise mechanism by which CPT regulates the IL-17 signaling pathway and improves liver diseases.

## 5. Conclusions

The results of the current study demonstrated that CPT could ameliorate CCl4-induced ALI by alleviating liver injury, inflammation, and oxidative stress. Integrative network pharmacology, transcriptomics, and pharmacological analyses revealed that CPT may be related to the TLR, TNF, and IL-17 pathways and the suppression of TNF-α, TLR9, and ADORA2B-mediated inflammation, oxidative stress, and apoptosis. These findings confirm that CPT affects ALI through multiple targets and multiple pathways, establishing a theoretical foundation for the application of CPT in the treatment of liver injury.

## Figures and Tables

**Table 1:** Information on the PCR primers.

Gene	Forward Primer	Reverse Primer
*β-Actin*	GTGCTATGTTGCTCTAGACTTCG	ATGCCACAGGATTCCATACC
*TNF-α*	ATGTCTCAGCCTCTTCTCATTC	GCTTGTCACTCGAATTTTGAGA
*TLR9*	ATGGTTCTCCGTCGAAGGACT	GAGGCTTCAGCTCACAGGG
*ADORA2B*	ATGCAGCTAGAGACGCAAGAC	GGGATACCAGAAAGTAGTTGGTG

## Data Availability

The authors confirm that the data supporting the findings and conclusions of this study are available in the article.

## Supplementary Materials

The following supporting information can be downloaded at: https://www.mdpi.com/article/10.3390/biom15101449/s1, Figure S1: Original Western Blot Figures.
