# Supplementary figures and images for: Mechanistic Insights into the Protective Effects of Cryptotanshinone Against CCl4-Induced Acute Liver Injury in Mice via Network Pharmacology and Transcriptomics
Source: Biomolecules. 2025 Oct 14;15(10):1449. doi: 10.3390/biom15101449 (PMC12563509; doi:10.3390/biom15101449)

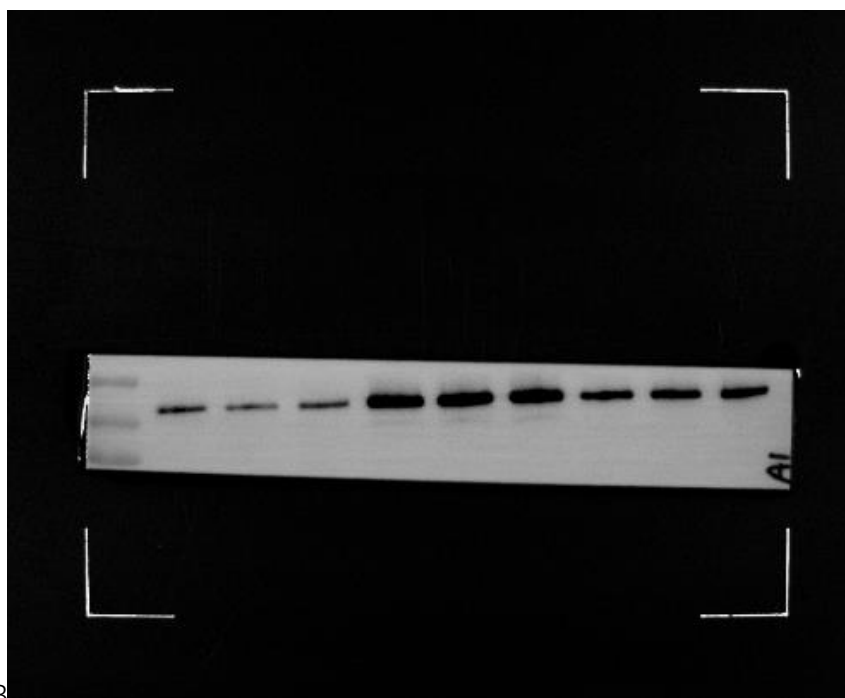

ADORA2B

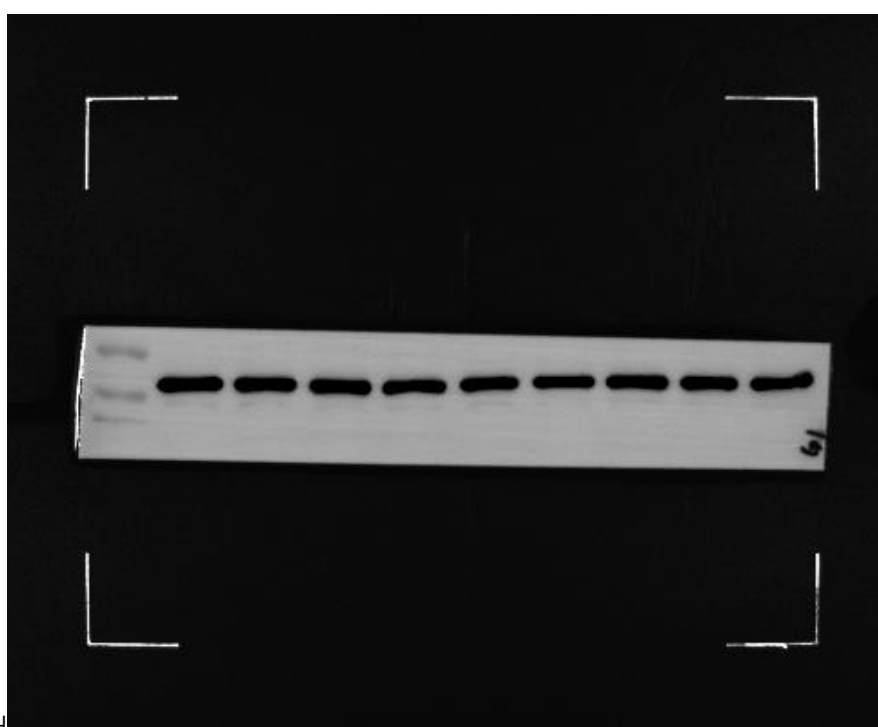

GAPDH

TLR9

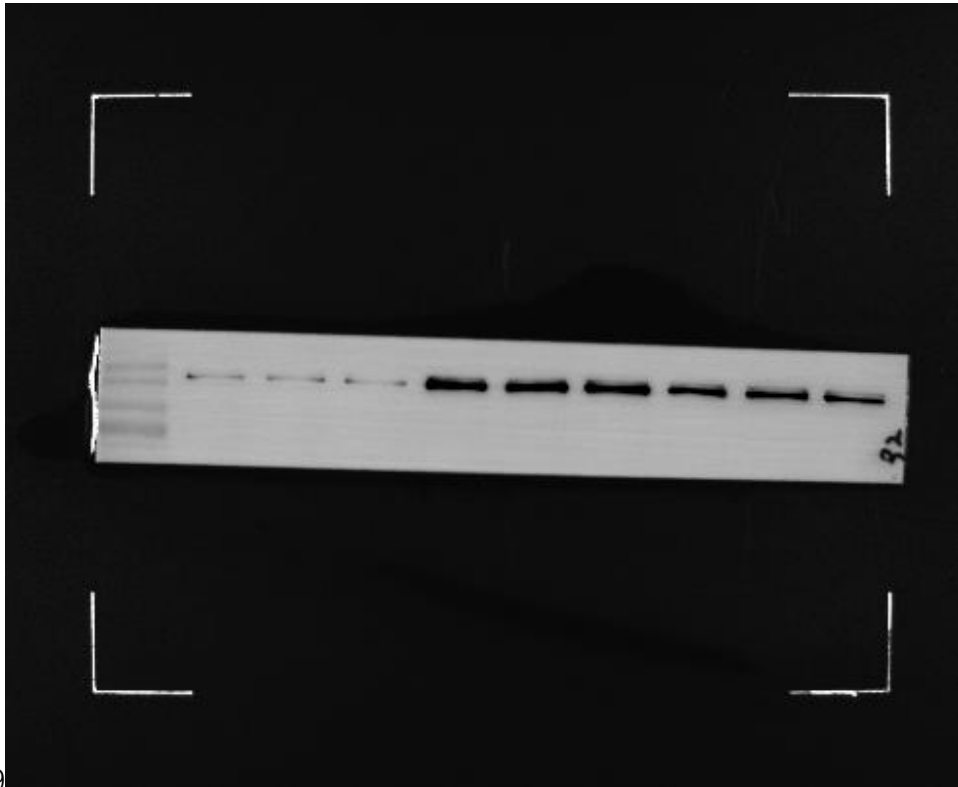

TNF $\alpha$

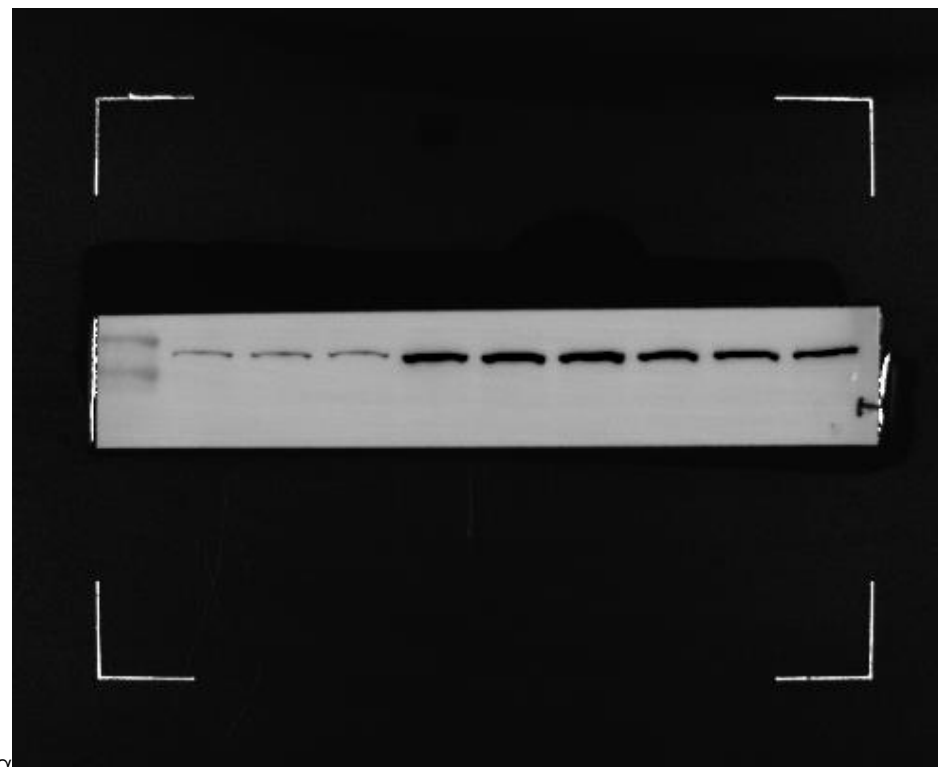

Supplement: Supplementary file 1 [file biomolecules-15-01449-s001.zip › biomolecules-3818546-supplementary.pdf]
